# The Impact of Risk-Adjusted Heparin Regimens on the Outcome of Patients with COVID-19 Infection. A Prospective Cohort Study

**DOI:** 10.3390/v13091720

**Published:** 2021-08-30

**Authors:** Pierpaolo Di Micco, Antonella Tufano, Giuseppe Cardillo, Egidio Imbalzano, Maria Amitrano, Corrado Lodigiani, Annamaria Bellizzi, Giuseppe Camporese, Antonella Cavalli, Carmela De Stefano, Vincenzo Russo, Antonio Voza, Alessandro Perrella, Paolo Prandoni

**Affiliations:** 1 Department of Medicine, Ospedale Buon Consiglio Fatebenefratelli di Napoli, 80122 Naples, Italy; 2 Department of Clinical Medicine and Surgery, Federico II University of Naples, 80131 Naples, Italy; atufano@unina.it; 3 Medylab, Clinical Chemistry, 81030 Lusciano, Italy; giuseppe.cardillo.75@gmail.com; 4 Department of Clinical and Experimental Medicine, University of Messina, 98124 Messina, Italy; egidio.imbalzano@unime.it; 5 Department of Medicine, AO Moscati, 83100 Avellino, Italy; amitranomaria@virgilio.it (M.A.); cdeste@gmail.com (C.D.S.); 6 Humanitas Clinical and Research Center IRCCS, 20089 Rozzano, Italy; corrado.lodigiani@humanitas.it (C.L.); antonio.voza@humanitas.it (A.V.); 7 Unit of Internal Medicine, Frangipane Hospital, 83031 Ariano Irpino, Italy; abellizzi1963@gmail.com (A.B.); antonella.cavalli@libero.it (A.C.); 8 Unit of Angiology, Department of Cardiac, Thoracic and Vascular Sciences, Padua University, 35100 Padua, Italy; giuseppe.camporese@aopd.veneto.it; 9 Chair of Cardiology, Department of Translational Medical Sciences, University of Campania Luigi Vanvitelli, 80131 Naples, Italy; v.p.russo@libero.it; 10 AO Cardarelli, 80131 Naples, Italy; alessandro.perrella@aocardarelli.it; 11 Arianna Foundation on Anticoagulation, 40138 Bologna, Italy; prandonip@gmail.com

**Keywords:** venous thromboembolism (VTE), SAR-CoV-2, pulmonary embolism, COVID-19

## Abstract

Background. According to recent guidelines, all hospitalized patients with COVID-19 should receive pharmacological prophylaxis for venous thromboembolism (VTE), unless there are specific contraindications. However, the optimal preventive strategy in terms of intensity of anticoagulation for these patients is not well established. Objectives. To investigate the impact of individualized regimens of enoxaparin on the development of VTE and on the risk of major bleeding complications during hospitalization in patients with COVID-19 infection. Methods. All consecutive patients admitted to the medical wards of six Italian hospitals between 15 September and 15 October 2020 with COVID-19 infection of moderate severity were administered enoxaparin in subcutaneous daily doses adjusted to the Padua Prediction Score stratification model: No heparin in patients scoring less than 4, 4000 IU daily in those scoring 4, 6000 IU in those scoring 5, and 8000 in those scoring six or more. Objective tests were performed in patients developing clinical symptoms of deep vein thrombosis and/or pulmonary embolism. Bleeding complications were defined according to the ISTH classification. Results. From the 154 eligible patients, enoxaparin was administered in all: 4000 IU in 73 patients, 6000 IU in 53, and 8000 IU in the remaining 28. During the course of hospitalization, 27 patients (17.5%) died. VTE developed in 14 of the 154 patients (9.1%; 95% CI, 4.6% to 13.6%), and was fatal in 1. Major bleeding complications developed in 35 patients (22.7%; 95% CI, 16.1% to 29.3%), and were fatal in 8. Conclusions. Despite the use of risk-adjusted doses of enoxaparin, the rate of VTE events was consistent with that reported in contemporary studies where fixed-dose low-molecular-weight heparin was used. The unexpectedly high risk of bleeding complications should induce caution in administering enoxaparin in doses higher than the conventional low ones.

## 1. Introduction

The novel coronavirus disease-2019 (COVID-2019) predisposes patients to venous thromboembolism (VTE) due to excessive inflammation, platelet activation, and endothelial dysfunction [1]. The risk of VTE is high, particularly in intensive care unit (ICU) patients, as shown by several observational studies [1,2]. According to recent guidelines, all hospitalized patients with COVID-19 should receive pharmacological VTE prophylaxis, unless there are specific contraindications [3,4,5,6,7,8]. However, the optimal prevention strategy in terms of dose and timing of administration is uncertain [9]. The available guidance documents differ in recommendations, with some suggesting prophylactic doses and others suggesting the swift to (sub)therapeutic doses, at least in patients at high risk for VTE [3,4,5,6,7,8].

In a prospective study, we investigated the incidence of objectively confirmed VTE and bleedings in patients admitted to medical wards with COVID-19 infection not severe enough to warrant hospitalization in ICU, as well as their association with the use of risk-adjusted preventive doses of low-molecular weight heparin (LMWH), established on individual basis according to the baseline value of the Padua Prediction Score (PPS) [10].

## 2. Materials and Methods

### 2.1. Patients and Thromboprophylaxis

**Figure 1:**
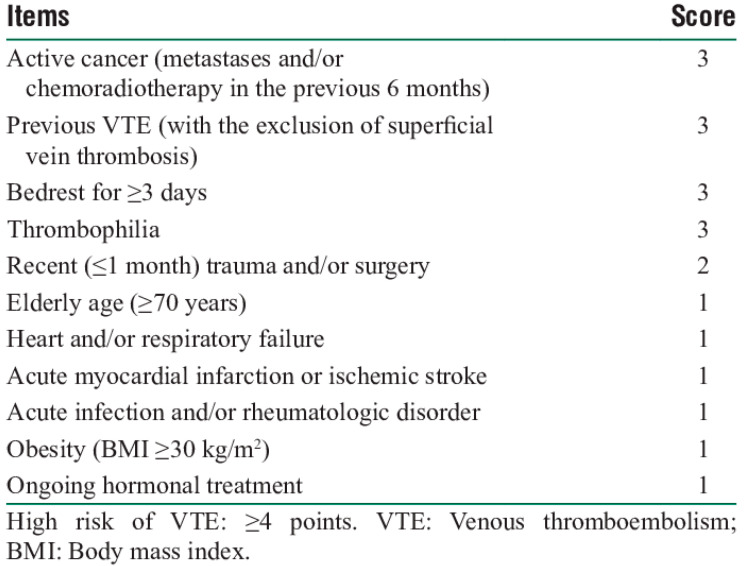
Padua Prediction Score. This picture is imported form the original paper added in reference number 10.

All consecutive patients admitted to the medical wards of six Italian Hospitals from 15th September to 15th October 2020 with laboratory-proven COVID-19 infection were eligible for inclusion in the present study. Patients who were taking anticoagulant therapy for any indication before COVID-19 diagnosis were excluded, as were those with previous VTE, those who had the diagnosis of VTE at referral, and those with contraindication to pharmacological thromboprophylaxis. The included hospitalized COVID-19 patients received VTE prophylaxis regimen according to the current International Guidelines [11,12]. Attending physicians were instructed to use the Padua Prediction Score (PPS) [10,11,12,13], as suggested by International Guidelines, to establish the need for thromboprophylaxis. PPS is reported in Figure 1. Patients with a score ≥4 received pharmacological prophylaxis with subcutaneous enoxaparin once daily, as follows: 4000 IU in those scoring 4, 6000 IU in those scoring 5, and 8000 IU in those scoring at least 6, irrespective of body weight (Table 1).

Patients were followed-up until hospital discharge, admission to ICU or death. The study was approved by the Institutional Ethical Board of each participating center, and patients gave their written informed consent for participation.

### 2.2. Assessment of Study Outcomes

At referral, routine clinical and laboratory parameters were recorded, including the main risk factors of VTE and the value of D-dimer. During hospitalization, all patients developing clinical symptoms of deep vein thrombosis (DVT) of the upper or lower extremities and/or pulmonary embolism (PE) underwent venous ultrasonography (US) and/or computed tomography pulmonary angiography (CT angiography). The development of major bleedings (MB) and clinically relevant non-major bleedings (CRNMB), defined according to the ISTH classification [14] was recorded, as was all-cause mortality.

### 2.3. Definitions

DVT was defined as a non-compressible venous segment during full compression involving the proximal veins and/or the below-knee axial veins. PE was defined as an intraluminal filling defect on spiral CT or pulmonary angiography.

Major bleeding (MB) was defined as fatal bleeding or symptomatic bleeding in a critical area or organ, or bleeding causing a fall in hemoglobin level of ≥2 g/dL or more or leading to transfusion of two or more units of whole blood or red cells. Clinical relevant non major bleeding (CRNMB) was defined as an overt bleeding not meeting the criteria for MB but requiring medical intervention [14].

### 2.4. Study Aims

The primary efficacy outcome was the composite of all events classified as DVT and/or PE occurring in the whole population during hospitalization. The primary safety outcome was the combination of MB and CRNMB occurring in the whole population during hospitalization.

All-cause mortality was a secondary outcome, as was the rate of thromboembolic and/or hemorrhagic complications occurring in each group of patients according to the different intensities of thromboprophylaxis.

Follow up was performed until 30 days after discharge.

### 2.5. Statistical Analysis

The rate (and the 95% CI) of symptomatic VTE and that of overall bleeding complications were calculated in the whole population, as well as in each of the three subgroups of patients, according to standard methods.

## 3. Results

### 3.1. Patients and Regimens of Thromboprophylaxis

Out of 240 consecutive eligible patients, 50 patients were excluded from the study because of ongoing anticoagulation, 30 because of contraindications in the use of anticoagulant drugs, and 6 because of refusal to give informed consent. Hence, 154 patients were recruited in the current investigation.

All patients received the assessment of the thrombotic risk according to the PPS. All 154 patients scored at least 4 of the PPS, and therefore qualified for LMWH prophylaxis. Based on the value of the PPS, 4000 IU of subcutaneous enoxaparin once daily were administered in 73 patients, 6000 IU in 53, and 8000 IU in the remaining 28.

### 3.2. Thromboembolic Complications, Bleedings and Deaths

Clinical characteristics of patients of studied cohort are summarized in Table 1. During hospitalization, 14 patients (9.1%; 95% CI, 4.6% to 13.6%) experienced VTE events (5 DVT, 1 SVT and 8 PE), which were fatal in 2; and 35 (22.7%, 95% CI, 16.1% to 29.3%) patients experienced MB and/or CRNMB, which were fatal in 8 cases (Table 2). The occurrence of VTE and bleeding at the same time was reported in 2 patients. Table 3 reports thrombotic events and/or bleedings in the three categories of enoxaparin prophylaxis. Clinical deterioration requiring admission to Intensive Care Units occurred in 15 patients (9.7%). Deaths from any reason were 27. The distribution of thrombotic and hemorrhagic events in different subgroups of patients that received thromboprophylaxis with different doses of enoxaparin has been reported in Table 3.

## 4. Discussion

Based on our results, the administration of enoxaparin in preventive doses adjusted to the baseline risk factors for VTE is unlikely to confer any appreciable advantage over the use of conventional fixed doses. Indeed, while the overall thromboembolic risk was consistent across the three categories of enoxaparin regimens, the risk of bleeding observed in patients assigned to the highest doses exceeded that reported among patients who were given lower doses. These findings are not surprising. Indeed, they are consistent with those of several recent investigations, which failed to show a favorable benefit/risk profile of intermediate doses of LMWH over the low conventional ones for protection against the thromboembolic risk in patients with COVID-19 infection [15].

Our results are robust, as they come from the prospective observation of consecutive patients recruited in a short time period at six hospital centers, a well-validated score (the PPS) was used to quantify the baseline thrombotic risk, and predefined stringent criteria were used for the adjudication of both thrombotic and bleeding complications. In spite of a limited sample size, the confidence intervals around the observed events rates make it unlikely to expect a more favorable prognosis by increasing the dosage of heparin prophylaxis in patients at a higher risk of VTE complications, as assessed with the PPS. Another study limitation is the lack of a control population assigned to fixed low doses of enoxaparin irrespective of the severity of risk factors for thrombosis.

Our findings provide further indirect evidence in support of the efficacy of enoxaparin for prevention of VTE events in patients hospitalized for a severe infectious disease. Indeed, despite the lack of a control population the rate of VTE complications we found in our patients (approximately 10%) is lower than that reported in patients managed without any thromboprophylaxis [16]. More significantly, heparin prophylaxis has been reported to improve the prognosis of patients with the COVID-19 disease remarkably, irrespective of the treatment dose [17,18]. As recent data suggest that therapeutic doses of heparin are likely to improve the prognosis of patients with moderate infection [19,20,21,22], in this category of patients the choice remains between therapeutic and prophylactic doses. Our findings suggest that tailoring the preventive doses of heparin according to the severity of risk factors for thrombosis does not confer an appreciable advantage in terms of benefit/risk ratio over the fixed doses.

## Figures and Tables

**Table 1:** Main baseline and clinical characteristics of the study patients in each of the three study groups.

	Enoxaparin 4000 U Daily PPS 4 (n = 73)	Enoxaparin 6000 U Daily PPS 5 (n = 53)	Enoxaparin 8000 U Daily PPS 6 or More (n = 28)
Active Cancer	5 (6.8%)	8 (15.1%)	2 (7.1%)
Hypomobility	42 (57.5%)	42 (79.2%)	22 (78.6%)
Previous Venous Thrombembolism	5 (5.6%)	4 (7.6%)	3 (10.7%)
Thrombophilia	1 (1.4%)	1 (1.9%)	0 (0%)
Recent Trauma or Surgery	6 (8.2%)	1 (1.9%)	1 (3.6%)
Age > 70 Years	32 (43.8%)	26 (49.1%)	13 (46.4%)
Males	44 (60.3%)	24 (45.3%)	19 (67.9%)
Cardiopathies	33 (45.2%)	21 (39.6%)	9 (32.1%)
Hormone Therapy	5 (5.6%)	6 (11.3%)	0 (0%)
BMI > 30	13 (17.8%)	12 (22.6%)	16 (57.1%)
Smoking	18 (24.7%)	10 (18.9%)	16 (57.1%)
D-Dimer > 1500 mg/L	12 (16.4%)	15 (28.3%)	7 (25.0%)

**Table 2:** Thrombotic and haemorrhagic events in the overall study cohort.

Venous Thromboembolism and Site of Evidence	Number of Patients
Fatal Pulmonary Embolism	2 (1.2%)
Non-Fatal Pulmonary Embolism	6 (3.8%)
Proxymal Deep Venous Thrombosis of Lower Limbs	3 (1.9%)
Distal Deep Venous Thrombosis of Lower Limbs	2 (1.2%)
Superficial Vein Thrombosis of Lower Limbs	1 (0.6%)
**Bleedings and Site of Bleedings**	
Gastrointestinal	1 (0.6%)
Cerebral	1 (0.6%)
Retroperitoneal	4 (2%)
Thoracic	3 (1.9%)
Intraocular	3 (1.9%)
Pericardial	2 (1%)
Muscolar Haematoma	8 (5.1%)
Anemia >2 g/dL Hb without Overt Bleeding	13 (8%)

**Table 3:** Distribution of thrombotic and/or haemorragic events in different subgroup of the analyzed cohort of inpatients with COVID-19.

Events	Patients	Clexane (U/day)		
		4000	6000	8000
Bleedings and Death	8	4	2	2
Bleeding and Venous Thrombosis	1	1	0	0
Bleeding and Pulmonary Embolism	1	0	1	0
Pulmonary Embolism amd Venous Thrombosis and Death	2	1	0	1
